# New Materials in Oral Surgery

**DOI:** 10.3390/ma13051034

**Published:** 2020-02-25

**Authors:** Vincenzo Grassia, Ludovica Nucci

**Affiliations:** Multidisciplinary Department of Medical-Surgical and Dental Specialties, University of Campania “Luigi Vanvitelli”, Via Luigi de Crecchio 6, 80138 Naples, Italy; ludortho@gmail.com

**Keywords:** biomaterials, tissue engineering, dental implants, oral rehabilitation bone materials

## Abstract

Currently, in the field of dentistry literature, one of most active research topics is clearly related to implants, bone materials, and regenerative strategies for the reconstruction of different oral tissues. Biomaterials and related technologies used with these purposes could only be derived from the integration of the knowledge of different disciplines, which together are skilled in generating innovation and research development, with extensive support of basic sciences and intense international cooperation. The combination of these resources, associated with the greater need for increasingly comprehensive and predictable therapeutic protocols, brings a substantial change in the treatment of oral rehabilitations.

## 1. Introduction

The development of biotechnologies and related new biomaterials is a major field of research and clinical innovation in all modern dentistry divisions, but especially so in oral surgery. In the current era of tissue engineering with the evolution of the properties, safety and efficiency of tissue regeneration and implantable materials, biomaterial research has contributed largely to the improvement in treatments strategy and to the suggestion of new therapeutic approaches in oral rehabilitation [1,2,3].

However, currently the most important research topic is clearly related to dental implants, bone materials, and regenerative medicine strategies for the reconstruction of oral tissues [4] These biomaterials and related technologies can only be developed through the cooperation between many clinical disciplines, with the extensive support of basic sciences and intense international cooperation [5].

The interdisciplinary field of implant-supported oral rehabilitation alone is generating considerable literature, probably the largest literature from all dental-related topics today [6,7]. This interdisciplinary field offers many perspectives to these treatments and how to improve them, often with slightly different perspectives. The scientific production related to these biomaterials is particularly intense, and sometimes confusing [8]. As a clinical discipline, dental implantology is at the border between oral and maxillofacial surgery, periodontology, and prosthodontics. The prosthodontics perspective participates in the improvement of material resistance and implant-supported treatment conception and planning. The periodontology perspectives offer adapted concepts in the management of peri-implant tissues, while the oral and maxillofacial surgery perspectives highlight the widest possibilities of tissue reconstruction or regeneration [9,10,11]

This interdisciplinary collaboration also extends far outside the limited dental field to most medical disciplines as biomaterials often have multiple potential applications in the human body. Dental implants, bone substitutes, surgical adjuvants, and other instruments and the way to combine them all into a successful therapeutic strategy, particularly represent a new frontier in regenerative medicine with numerous applications in orthopedic, plastic surgery, and other related medical disciplines [12,13].

Today, the field of dental implants and related biomaterials is also the heart of translational research in dentistry. All of the new biomaterials are thoroughly designed and tested by material scientists, before they are finally tested in vitro, in vivo, in animal models, and finally in clinical settings. The translational research movement is always in both directions: basic to clinical sciences and vice versa [14,15,16,17]

The international cooperation is an important step to develop and spread new ideas and projects, promote debate, contradiction, and sometimes consensus, and finally grow a shared literature with less misunderstandings, confusion, and bias [18].

One final aspect of this field is related to the social sciences and appeared quite recently with the considerable development of this scientific field: the internationalization of higher education and research in this domain, its importance, and its consequences. With the growth of the scientific field and its industrial development, implant dentistry step by step became a major discipline worldwide in oral and maxillofacial sciences, leading to the constitution of various national leaderships and centers of research and education [7,14,15].

In this special issue on new materials in oral surgery and implant dentistry, we continued our task to gather a meaningful corpus of relevant articles. More than before, a better control of the specialized literature is due. We particularly describe the strengths, weaknesses, opportunities, and threats of these disciplines and how research in implantable materials, especially dental implants (new implant design and surfaces), bone materials, or surgical adjuvants, is affected by not only scientific bias and misunderstandings, but also industrial and financial interferences, creating many inaccuracies in the literature. Despite the incredible potential and revolution that these disciplines are supporting for patients and the clinical approach to oral rehabilitation, there is also a major threat to the credibility of this field [19,20].

We selected a series of articles with new data on a wide range of topics in biomaterials and regenerative research in periodontology, oral surgery, and aesthetic and implant dentistry, and we highlight how these interconnected fields of research are both transversal (multidisciplinary) and translational (from basic sciences to clinical applications). In the following articles, most of the major current aspects of biomaterials in oral surgery are introduced.

Several articles have focused on the molecular, cellular, and pharmaceutical aspects of the biomaterials used for bone grafting. More specific aspects have been investigated by other authors such as the biological mechanisms of dental implants, insisting in particular on the impact of implant surface topography on peri-implant tissues.

Therefore, the articles gathered for this Special Issue highlight only a selection of key aspects of the current dental biomaterial sciences, which are both founding knowledge and innovative perspectives for the future of the field.
